# Rural-Urban Migration, Childbearing Decision-Making, Fertility and Contraceptive Perspectives of Street Adolescents and Youth in Kampala, Uganda

**DOI:** 10.3389/frph.2022.869118

**Published:** 2022-07-29

**Authors:** Mulekya Francis Bwambale, Cheryl A. Moyer, Paul Bukuluki, Bart van den Borne

**Affiliations:** ^1^Faculty of Health, Medicine and Life Sciences, Care and Public Health Research Institute (CAPHRI), University of Maastricht, Maastricht, Netherlands; ^2^Global Reach, Department of Medical Education, University of Michigan Medical School, Ann Arbor, MI, United States; ^3^Department of Social Work and Social Administration, School of Social Sciences, Makerere University, Kampala, Uganda

**Keywords:** rural-urban migration, street adolescents and youth, childbearing decision-making, contraceptive and fertility intentions, Uganda

## Abstract

**Introduction:**

This paper aims to describe and assess social demographic factors associated with childbearing decision-making, fertility and contraceptive intentions among street adolescents and youth in Kampala, Uganda while considering rural-urban migration as an explanatory factor.

**Materials and Methods:**

A cross-sectional survey of 513 adolescents and youth aged 12–24 years self-identifying as street adolescents and youth were interviewed with a structured questionnaire in 2019. Street adolescents and youth who migrated from other rural districts to Kampala were compared with those from the city. Logistic regression was performed to assess associations between the independent factors and personal childbearing decision-making, fertility and contraceptive intentions.

**Results:**

Overall, 80.31% of the street adolescents and youth had a rural-urban migration experience. Fifty six percent (56.32%) of the street adolescents and youth made personal childbearing decisions, 94.15% expressed intentions to have children in the future and 42.88% expressed intentions to use contraceptives in the future. Intentions to use contraceptives were significantly higher among males (58.75%) than females (20.00%), and higher among migrants (65.91%) compared to non-migrants (34.09%). Contraceptive intentions were positively associated with self-perceived permanent residential status (aOR = 10.26, 2.70–39.08), intra-urban mobility (aOR = 4.99, 95%CI 1.50–6.59) and intentions to migrate to other towns within the country (aOR = 5.33, 95%CI 1.59–17.80). Being married (aOR = 0.13, 95%CI 0.02–0.85), a large shelter population size (aOR = 0.13, 95%CI 0.03–0.63) and having repeat migrations between the city and home district (aOR = 0.23, 95%CI 0.05–0.94), including migration-associated challenges reduced the odds of street youth's personal childbearing decision-making, while belonging to a social support group increased the odds of childbearing decision-making. We found no significant association between social demographic characteristics and fertility intentions.

**Discussion:**

Factors that influenced personal childbearing decision-making and contraceptive intentions among street adolescents and youth in Kampala operate mainly at the interpersonal and community levels, with marital status, shelter population size, rural-urban migration and its associated challenges associated with childbearing decision-making. Interventions to promote childbearing decision-making and contraceptive use among street adolescents and youth should take into consideration their migration and intra-urban mobility patterns.

## Introduction

In sub-Saharan Africa (SSA), fertility rates have been found to be more relatively high in comparison to the developed countries and regions. Similarly, the decline in fertility trends in SSA is lower than other regions (1, 2). The persistence of high fertility in SSA is due to high levels of poverty and insecurity as well as weaknesses in health care systems to address the unmet need for family planning and other basic services (3). Precious studies found that the average number of years of primary schooling among the adult population, rather than income standards, child mortality, or total mortality rates, drive fertility down by about 40–80% when those years grow from zero (no illiteracy) to six years (full literacy) (3, 4). It is possible that some countries in SSA appear exceptional simply due to the much lower levels of education (5). Given that SSA is the world's fastest growing region, with a population projected to more than double by 2050, it is essential to understand the perspectives of fertility and contraceptive use among vulnerable youth. Concerns about high fertility among youth might predict low contraception uptake (6). Literature on use of contraceptives and willingness to use them among homeless youth in SSA is limited. Systematic reviews shows that contraceptive use among women aged 15–49 years with no fertility intention in sub-Saharan Africa remains low at 29.6%, with cross-country variations (7). Studies done in the United States of America showed that 80% of the women used a condom while 73% of homeless teenagers had willingness to use contraceptives (8). Studies in East Africa have focused on knowledge, attitudes and access to contraceptive use and fertility intentions mainly among women and HIV clients, and many have been predominantly qualitative in nature (9, 10). Fertility desires were found to be similar among antiretroviral therapy service users and non-users with more than 60% of women in Uganda interested in using long acting contraceptives (11, 12). These studies quantify the use of contraceptives to be low and higher among married adolescents (9).

While fertility has remained at the pre-transition level in Uganda over the past 20 years, there are signs of fertility decline with specific groups of women especially the most educated, urban and those in the Eastern region (13). Despite the declining fertility trends over the past decades, Uganda continues to record a persistently high total fertility rate and high adolescent pregnancy rate of 5.4 and 33%, respectively (14, 15). In addition, Uganda has one of the highest annual population growth rates in the world estimated at 3% per annum. In spite of the high total fertility rates, fertility desires in Uganda have remained high in rural and high HIV burden contexts, with rural men (69.9%) wanting to have more children in the future than women (57.1%) and were associated with an unfilled desired family size, being male and primary education (16). About 35% of married women use modern contraception and 28% have an unmet need for family planning (17). While 25% of women age 15–19 have begun childbearing, modern contraceptive use among married and adolescents is generally low at 9.4% (9, 10). The persistent low contraceptive use and high teenage pregnancy rate have been attributed to sexual practices, limited access to contraception, perceptions and inadequate knowledge on contraceptives and fear of side effects (18). Among street adolescents and youth, our previous work revealed that 16% of migrants used modern contraceptives compared to 27.72% of their non-migrant counterparts (19). Given the high unmet needs and low prevalence of family planning services use, knowing if urban street young people have any intentions to use contraceptives or want to have children in the future is worth investigating.

In Uganda, youth migration from rural to urban environments has resulted in an explosion of youth living in dynamic and informal city settlements (20). Descriptive analyses suggest that the decline in migrant fertility could be related to the rapid and pronounced improvement in standard of living experienced by migrants after settling in the urban area, and may also be due in part to temporary spousal separation (21). In spite of the influence of migration on fertility, much less attention has been devoted to the relationship of rural-urban migration with regard to fertility intentions and contraceptive desires. Available literature suggests that migration may induce a change in how individuals make decisions about one important events, such as when and whether to have a child (22). To date, the majority of research on knowledge, concerns and determinants of contraceptive use and fertility services has been focused on women and HIV clients (23–25). To our knowledge, there is a dearth of literature on urban street youth's sexual and reproductive health choices in regard to childbearing decision-making, contraceptive and fertility perspectives. The focus on the street adolescents and youth is premised on the global call for universal sexual and reproductive health (SRH) coverage and improvement in urban health, leaving no one behind (26). Moreover, street children and youth in cities are among the marginalized and hard-to-reach populations, and are often missing in national population censuses and surveys as the temporary shelters in which they stay may not be included (3, 27).

This study aimed to assess childbearing decision-making, fertility intentions and contraceptive perspectives, and associated social demographic factors, among disadvantaged street adolescents and youth in Kampala, Uganda while considering rural-urban migration as an explanatory factor. Furthermore, analyses are performed to understand how these relationships may operate differently for migrants and non-migrant street youth and age groups. Understanding childbearing decision-making and fertility intentions of street adolescents and youth can be a powerful predictor of their contraceptive behavior. Knowledge of the migration intentions of street adolescents and young people is important in predicting future actual migration and its impact on future fertility rates. Lastly, this important study might have implications in framing future family planning policies and strategies to improve the sexual and reproductive health of urban street adolescents and youth, who are often disregarded in urban policies and plans (28).

## Materials and Methods

### Study Design, Study Population and Sampling

This study used data from a cross-sectional survey of 513 street adolescents and youth living in Kampala, Uganda that was conducted between April and July 2019. Detailed methods have been described in our previous work (29), but will be briefly reviewed in this section. The study was conducted in three of five divisions of the Kampala capital city, including Makindye, Rubaga and Central divisions. Eligible respondents included adolescents and youth aged 12–24 years who identified themselves as street adolescents and youth. The sample size was calculated in STATA (12.0, TX USA) using the formula [sampsi.25.35, alpha (0.05) power (0.9) one sample] for a two-sided hypothesis and reference proportion (p0) of 25%–the proportion of adolescents who reported having had more than one sexual partner in western Kenya (30). The sample size was adjusted upwards to cater for a 20% non-response rate and design effect of two.

To select the sampling units, probability proportional to size sampling was applied based on parish census population size estimates. Geographic locations/venues where street adolescents and youth congregated during daytime were identified through the pre-survey mapping exercise. The two questions which were asked to identify street youth were: In which place/location within this city do you reside? Where do you normally spend most of your time during the day for a livelihood? Those who mentioned geographic locations which were known as congregation areas or places of stay for street youth and who spent most of their time during the day on the streets were considered street youth. Venue-based time-space (VBTS) sampling technique was applied to select respondents for the interview. All interviews were conducted in private spaces on the streets by a team of 14 trained research assistants using a pre-tested semi-structured questionnaire. The questionnaire covered topics such as demographics, migration history, sexual and reproductive health decision making, service utilization and fertility and contraceptive intentions and was configured for data collection on tablet computers. This was aimed to minimize non-response and improve the internal validity of the responses.

To further minimize non-response and improve the internal validity of the responses, the questionnaire was translated into two local languages, *Luganda* and *Ngakaramajong*, which are commonly spoken by street youth. Access to the respondents was made possible through the street youth peer networks, their caregivers and local urban zonal leaders who served as guides during data collection. In compensation for their participation in the study, street adolescents and youth were rewarded with an incentive of UGX 2000 (USD 0.57).

### Measurements

The main study outcomes included personal childbearing decision-making, fertility intentions, and contraceptive intentions. Fertility intentions were conceptualized as the desire to have children in the future, irrespective of whether the street youth had ever had children or not, and were assessed by asking two consecutive questions: Have you ever become pregnant/had children in your lifetime? (Yes/No). Those who responded in the affirmative were further asked: Do you have any plans to have children in the future? (Yes/No). A “Yes” response to the latter question was considered as fertility intentions. In addition, fertility intention among sexually active adolescents and youth who had had children in their lifetime were assessed. Contraceptive intentions were conceptualized as the desire to use modern contraceptives in the future and were measured by asking the question: Do you have any plans to use any modern family planning method in the future? (Yes/No). This was followed by a question that enlisted the types of contraceptive methods that street youth preferred to use in the future. Childbearing decision-making was defined as the ability of a street youth to make her or his own decisions regarding the number of children he or she wanted, and was assessed by asking the question: Who makes decisions for you regarding the number of children you want/would like to have in the future? (self, partner, my partner and I, parents/in-laws, friends/peers, other). A “self” response to this question was considered as personal childbearing decision making.

Rural-urban migrants were defined as street adolescents and youth who migrated from other districts and regions of Uganda to Kampala capital city, irrespective of their reason for migration. Elsewhere in the African region, migrants have been found to face challenges that negatively affect their health, including poor accommodation and social safety, and low levels of social support in urban destination communities, and hence a complex systems approach to understanding the sexual health of migrants is warranted (31). Rural-urban migrant street youth who reported having had more than one repeat or return migrations between the city and district of origin were further classified as circular migrants. Other aspects of migration measured in this study included intra-urban mobility–was defined as the number of places stayed following migration to the city and respondents were classified as non-mover (one place or no move) or mover (two or more places moved). Previous studies on intra-urban residential mobility of city residents in East Africa have applied similar categorisations (32, 33).

### Data Analysis

Statistical analysis was conducted in Stata (version 15). At analysis, respondents with rural-urban migration experience were classified as migrants while those without the rural-urban migration experience were considered as non-migrants. Other aspects of migration measured included repeat movements between the city and districts of origin and intra-urban mobility. Descriptive characteristics of the respondents are presented as frequencies and percentages. For bivariate analyses, crosstabulations and logistic regression were performed to establish associations with childbearing decision-making, contraceptive and fertility intentions by migration status and sex. Associations were tested using Pearson Chi-Square (x2) tests. Statistical significance was obtained using 95% confidence interval (CI) at *p* ≤ 0.05. Multivariate logistic regression analyses were conducted on the three binary outcomes of interest (childbearing decision making, fertility and contraceptive intentions). All factors significant at the *p* ≤ 0.05 at the bivariate analysis including migration status and known predictors of the three study outcomes were entered into the final binary logistic regression models as shown in **Tables 2–4**.

### Ethical Considerations

The study protocol and all study materials were reviewed and approved by the Makerere University School of Social Sciences Research and Ethics Committee (Ref. MAKSS REC 12.18.389). Informed written consent was obtained directly for respondents aged 18 years or older. Both caregiver/guardian consent and informed assent were obtained for respondents 17 years and younger, guided by the Uganda National Council of Science and Technology (UNCST) guidelines on conducting research with human subjects. Written informed consent and assent were obtained prior to the interview.

## Results

### Background Characteristics

The total sample size was 513 street adolescents and youth: 303 males (59.06 %) and 210 females (40.94%). Overall, respondents had a mean age of 18.5 years, however female respondents were older (mean age 20.40 years) than male respondents (mean age 15.00 years). The median sexual debut was 17 years, 16 years for females and 17 years for males. Overall, more than a third of the females (37.14%) compared to a low percentage of males (2.64%) were in conjugal relations. Approximately 16.76% (*n* = 86) of the street adolescents and youth reported that they were in conjugal relationships. Regarding living arrangements, 12.09% of the street adolescents and youth stayed alone, 57.31% stayed with a partner or friend, 10.9% stayed with parents (both parents, mother, and father alone) while 19.69% stayed with siblings/other relatives. Irrespective of age, the mean number of children per street youth was two children (x = 1.67, SD 0.83) for females and one child (x= 1.24, SD 0.44) for males, and the range was 1–4 children.

Regarding migration status, 80.31% of the street adolescents and youth had migrated to the city while 19.69% were non-migrants. Circular migration, defined as having more than one repeat migration between Kampala capital city and district of origin was found to be frequent. Almost half (51.76%) of migrant street adolescents and youth had more than one repeat migration to the district of origin in the 24 months prior to the survey, with a median of two (2) repeat migrations [IQR = 7 (Q1-Q=1, Q3=8)]. About three quarters (71.06%) of the street adolescents and youth had intentions to return to their districts of origin; 45.22% desired to relocate to another space within the city while 34.70% desired to migrate to another urban center within the country.

### Childbearing Decision-Making

About half (56.32%) of the street adolescents and youth expressed making own personal childbearing decisions, (28.57%) made joint decisions with a sexual partner and 15.11% involved other people - parents, friends, in-laws, and God (religious beliefs), in childbearing decision making. To establish if street adolescents and youth and their sexual partners' childbearing preferences were in agreement, respondents were asked if they wanted the same number of children, more or fewer than their sexual partners. Overall, 15.11% (*n* = 73) of the street adolescents and youth wanted the same number of children as their partners, 8.70% (*n* = 42) wished to have more children, 12.42% (*n* = 60) desired fewer children while 63.77% were undecided on the number of children. Less than three-quarters (71.23%) of the male street adolescents and youth made personal childbearing decisions compared to 34.85% of their female counterparts. Table 1 shows the distribution of childbearing decision-making, fertility and contraceptive intentions and background characteristics. About three quarters (75.74%) of migrant street youth made personal childbearing decision-making compared to their migrant counterparts (24.26%)(χ2 = 8.3239; α = 0.004).

**Table 1:** Crosstabulation of socio-demographics, childbearing decision-making, fertility, and contraceptive intentions among street adolescents and youth by sex and migration status, Kampala, Uganda, 2019.

**Characteristic**	**Male** ***n* (%)**	**Female** ***n* (%)**	**χ2** **(p/t-test)**	**Non-migrant** ***n* (%)**	**Migrant** ***n* (%)**	**χ2** **(p/t-test)**
**Age (in complete years)**			1.30 (0.739)			1.01 (0.312)
12–14	37 (12.21)	20 (9.52)		10 (9.90)	47 (11.41)	
15–17	80 (26.40)	58 (27.62)		24 (23.76)	114 (27.67)	
18–20	102 (33.66)	77 (36.67)		33 (32.67)	146 (35.44)	
21–14	84 (27.72)	55 (26.19)		34 (33.66)	105 (25.49)	
Mean age (SD)	15.22 (1.53)	20.44 (2.02)		19.04 (3.23)	18.31 (3.09)	
**Marital status**			105.817 (<0.001)			12.578 (<0.001)
Not married	295 (97.36)	132 (62.86)		96 (95.05)	331 (80.34)	
Married/cohabiting	8 (2.64)	78 (37.14)		5 (4.95)	81 (19.66)	
**Childbearing decision making**			62.852 (<0.001)			8.32 (0.004)
Other	82 (28.77)	129 (65.15)		29 (30.53)	182 (46.91)	
Self	203 (71.23)	69 (34.85)		66 (69.47)	206 (53.09)	
**Fertility intentions**			0.012 (0.914)			0.00 (0.965)
No	18 (5.94)	12 (5.71)		6 (5.94)	24 (5.83)	
Yes	285 (94.06)	198 (94.29)		95 (94.06)	388 (94.17)	
**Contraceptive intentions**			76.02 (<0.001)			50.54 (<0.001)
No	125 (41.25)	168 (80.00)		26 (25.74)	267 (64.81)	
Yes	178 (58.75)	42 (20.00)		75 (74.26)	145 (35.19)	
**Median sexual debut (IQR)**	16 (8–21)	17 (12–20)		16 (10–20)	17 (7–21)	

### Fertility Intentions

First, we asked if both male and female street adolescents and youth had ever had any children in their lifetime. Overall, 64.06% of the female respondents reported they had ever become pregnant compared to 33.54% of the males who had children or impregnated others in their lifetime. The number of children were 1–4 for females compared to 1–2 for males. At the time of the survey, 8.10% (*n* = 17) of the female street adolescents and youth were pregnant. Among those who were pregnant, 88.24% (*n* = 15) were married and 82.35% (*n* = 14) were aged 18 years or older. Older street youth (18–24 years) had significantly higher fertility intentions (63.15%) compared to 36.85% of the younger counterparts (12–17 years) (χ2 = 4.7060; α = 0.030). Second, we assessed fertility intentions among street adolescents and youth by asking them about their plans to have children in the future. Overall, 94.15% of the street adolescents and youth expressed intentions to have children in the future, irrespective of the duration or number of children. Among the females, 84.76% (*n* = 178) affirmed their desire to have children in the future, 1.43 (*n* = 3) had intentions to become pregnant now, 8.10% (*n* = 17) were currently pregnant while 5.71% (*n* = 12) reported no firm intentions to have children in the future.

### Contraceptive Intentions

Both contraceptive use and intentions to use modern contraceptives in the future among street adolescents and youth were assessed. Actual modern contraceptive use among street adolescents and youth was found to be low at 18.13%. The most preferred contraceptive was a condom (75%) followed by an injectable (11.36%), intra uterine device (8.64%), oral contraceptive (3.64%) and Norplant (1.36%). Overall, less than half (42.88%) of the street adolescents and youth expressed intentions to use contraceptives in the future. Contraceptive intentions were not significantly higher among older street youth 64.09% compared to the younger age group 12–17 years (35.91%) (χ2 = 0.7227; α = 0.395). Findings from Table 1 show that, overall, 58.75% of the male street youth expressed contraceptive intentions compared to 20.00% of the female counterparts. About two thirds of migrant street youth expressed contraceptive intentions compared to a third of their non-migrant counterparts. Among the migrant street youth, desire for contraceptive use in the future was higher among non-circular migrants (85.52%) compared to circular migrants (14.48%).

## Factors Associated With the Three Outcomes

### Childbearing Decision-Making

At the bivariate level, the majority (61.03%) of the older street youth (18–24 years) made personal decisions regarding childbearing compared to 38.97% of the younger counterparts (12–17 years) and the difference was not statistically significant (χ2 = 0.1997; α = 0.273). Table 2 shows the results of the bivariate and multivariate models with childbearing decision-making as the primary outcome. The odds of personal childbearing decision making were 87% lower if street adolescents and youth were in conjugal relation compared to those in non-conjugal relations (aOR = 0.13, 95%CI 0.02–0.85). Similarly, the odds of personal childbearing decision making were 87% lower if the mean number of street adolescents and youth living in a shelter was more than five occupants compared to those with fewer shelter occupants (aOR = 0.13, 95%CI 0.03–0.63). The odds of personal childbearing decision-making were 50% lower if the street adolescents and youth were migrants compared to the non-migrants (cOR = 0.50, 95%CI 0.31–0.81). In fact, circular migrants were 54% less likely to make own childbearing decisions compared to non-circular migrants (cOR = 0.46, 95%CI 0.30–0.72). Migrant street youth were 50% less likely to make own childbearing decisions than their non-migrant counterparts (cOR = 0.50, 95%CI 0.31–0.81).

**Table 2:** Model 1–Binary logistic regression of migration aspects and social demographic factors with personal childbearing decision-making among street adolescents and youth in Kampala, Uganda, 2019.

**Explanatory factors**	**Bivariate model** **cOR (95%CI)**	**Multivariate model** **aOR (95%CI)**
Age in years (12–17 = 0, 18–24 = 1)	0.81 (0.56–1.18)	0.79 (0.228–2.719)
Sex (male = 0, female = 1)	0.22 (0.15–0.32)	0.63 (0.130–2.917)
Education attained (primary = 0, secondary +tertiary = 1)	1.86 (1.19–2.90)	2.60 (0.750–9.00)
Marital status (not married = 0, married = 1)	0.22 (0.13–0.37)	0.13 (0.02–0.85)
Mean shelter occupants of five people (≤ mean = 0, >mean = 1)	0.42 (0.28–0.66)	0.13 (0.03–0.63)
Religion (other = 0, Christian = 1)	0.60 (0.38–0.95)	0.73 (0.18–2.88)
Residential status (Mobile = 0, permanent = 1)	2.82 (1.91–4.17)	0.546 (0.13–2.36)
Sex debut (<18 yrrs, 18 years and above = 1)	0.43 (0.26–0.71)	2.824 (0.66–12.07)
Intra-urban mobility (non-mover = 0, mover = 1)	2.36 (1.58–3.54)	0.74 (0.22–2.47)
Circular migrant (no = 0, yes = 1)	0.46 (0.30–0.72)	0.23 (0.05–0.94)
Experienced challenges during migration (no = 0, yes = 1)	0.30 (0.19–0.47)	0.20 (0.05–0.72)
Intention to migrate to another town (no = 0, yes = 1)	2.09 (1.42–3.08)	0.53 (0.15–1.82)
Belongs to a support group (no = 0, yes = 1)	1.24 (0.76–2.04)	4.66 (1.30–16.63)
Source of social support (other = 0, friends = 1).	0.64 (0.45–0.92)	0.66 (0.24–1.87)
Migration status (non-migrant = 0, migrant = 1)	0.50 (0.31–0.80)	0.29 (0.01–6.71)

After controlling for cofounding, only a few variables, that is, marital status, mean shelter population size, circular migration aspects and belonging to a support group remained statistically significant in the final multivariate model. Street adolescents and youth who were in conjugal relationships were 87% less likely to make own decisions regarding childbearing than the non-married (aOR = 0.13, 95%CI 0.12–0.85). Similarly, odds of personal childbearing decision making were reduced if street adolescents and youth size was more than a mean population size of five occupants compared to those staying in shelters with fewer people (aOR = 0.13, 95%CI 0.03–0.63). The odds of personal childbearing decision-making were 74% lower if the street adolescents and youth had repeated movements in the 24 months prior to the survey compared to non-repeat movers (aOR = 0.25, 95%CI 0.07–0.968). Similarly, the odds of personal childbearing decision-making were reduced by 80% if street adolescents and youth experienced challenges during the migration process compared to those that did not (aOR = 0.20, 95%CI 0.55–0.72). Belonging to a social support group increased the likelihood of personal childbearing decision-making four-fold among street adolescents and youth compared to those who had no belonging to a social support group (aOR = 4.25; 95%CI 1.21–14.96). Notably, age and sex did not remain significant in the adjusted model.

### Contraceptive Intentions

Table 3 shows the results of the bivariate and multivariate models with contraceptive intentions as the primary outcome. Overall, female street adolescents and youth were 82% less likely to express the desire to use contraceptives than their male counterparts (cOR = 0.18, 95%CI 0.12–0.26). Street youth who had attained secondary and tertiary education were 3.28 times more likely to express contraceptive intentions compared to those who had attained primary education. No association was found between contraceptive intentions and different age groups (cOR = 3.28, 95%CI 2.14–5.04). Street adolescents and youth with intentions to migrate to another town within the country were 5.72 times more likely to have future contraceptive desires (cOR = 5.51, 95%CI 3.84–8.41). A similar finding was observed for those wanting to migrate to another country (cOR 5.51, 95%CI 3.77–8.08). The odds of contraceptive intentions increased six-fold for the street adolescents and youth who had intentions to migrate outside the country than those who did not want to migrate (cOR = 6.25, 95%CI 4.12–9.50). The odds of contraceptive intentions were 0.61% lower among circular migrant street youth in circular rural-urban movement than non-circular migrants (cOR = 0.29, 95%CI 0.17–0.48). Intentions to use contraceptives varied significantly by migration status. Non-migrants were 7.73 times more likely to express desire to use contraceptives in the future compared to the migrants (OR = 7.34, 95%CI 4.17–12.91). Similarly, migrants were 90% less likely to express desire for use of contraceptive in the future (cOR = 0.19, 95%CI 0.12–0.31).

**Table 3:** Model 2–Binary logistic regression of migration aspects and social demographic factors with contraceptive intentions among Street adolescents and youth in Kampala, Uganda, 2019.

**Explanatory variables**	**Bivariate model** **cOR (95%CI)**	**Multivariate model** **aOR (95%CI)**
Age in complete years (12–17 = 0, 18–24 = 1)	1.17 (0.82–1.68)	1.14 (0.27–4.84)
Sex (male = 0, female = 1)	0.18 (0.12–0.26)	0.331 (0.05–2.06)
Education attained (primary = 0, secondary +tertiary = 1)	3.28 (2.14–5.04)	0.926 (0.27–3.22)
Marital status (not married = 0, married = 1)	0.27 (0.16–0.48)	4.130 (0.50–34.31)
Mean shelter occupants (≤ mean = 0, >mean = 1)	0.24 (0.15–0.40)	0.300 (0.06–1.52)
Religion (other = 0, Christian = 1)	0.27 (0.17–0.42)	0.483 (0.11–2.09)
Residential status (Mobile = 0, permanence = 1)	8.54 (5.70–12.80)	10.264 (2.70–39.08)
Sex debut (<18 years = 0, 18 years and above = 1)	0.48 (0.29–0.80)	2.291 (0.54–9.75)
Intra-urban mobility (non-mover = 0, mover = 1)	4.58 (2.93–7.17)	5.00 (1.50–16.59)
Circular migrant (no = 0, yes = 1)	0.28 (0.17– 0.48)	0.99 (0.25–3.94)
Experienced challenges during migration (no = 0, yes = 1)	0.68 (0.45–1.05)	0.85 (0.25–2.91)
Intention to migrate to another town (no = 0, yes = 1)	5.72 (3.84–8.51)	5.33 (1.59–17.80)
Belongs to a support group (no = 0, yes = 1)	2.56 (1.59–4.12)	1.32 (0.37–4.77)
Source of social support (other = 0, friends = 1)	0.57 (0.40–0.81)	0.73 (0.27–2.21)
Migration status (non-migrant = 0, migrant = 1)	0.19 (0.12–0.31)	4.33 (0.23–80.48)

In the final multivariate model, intentions to use contraceptives in the future were positively associated with self-perceived permanent residential status, intra-urban mobility, and intentions to migrate. Street adolescents and youth who considered themselves to be permanently residents of the city were 10.26 times more likely to express intentions for contraceptive use than those who perceived themselves to be mobile residents (aOR = 10.26, 2.700–39.08). Intra-urban mobility, defined as having moved or stayed in more than one place since migrating to the city, increased the likelihood of contraceptive intentions by five-hold among movers compared to non-movers (aOR = 4.99, 95%CI 1.50–16.59). Similarly, street children with intentions to migrate to other towns within the country were 5.33 times more likely to likely to express contraceptive intentions than those without migration intentions (aOR = 5.33, 95%CI 1.59–17.80).

### Fertility Intentions

Table 4 shows the results of the bivariate and multivariate models with fertility intentions as the primary outcome. The fertility intentions of street adolescents and youth fertility intentions were associated with being married (cOR = 3.47, 95%CI 2.11–5.71), 18 years or older (cOR = 2.87, 95%CI 1.88–4.37), having attained primary or secondary (cOR = 1.95, 95%CI 1.27–2.98), earning a daily income of more than 1USD (cOR = 1.67, 95%CI 1.07–2.61) and belonging to a support group (cOR = 2.04, 95%CI 1.26–3.33). The odds of fertility intentions were 54% lower if the street adolescents and youth had first sex in life before age 18 than the adult street youth (cOR = 0.46, 95%CI 0.28–0.77). A sub-analysis of sexually active street youth and who already had children in their lifetime (parity 1–4 children) showed that the younger age group were 41.73 times more likely to express intentions for more children in the future than the older age group (18 years and above) (cOR = 41.73, 95%CI 10.15–171.52). Similarly, female street youth were 6.77 times more likely to express fertility intentions compared to their male counterparts (cOR = 6.77, 95%CI 4.06–11.27). Being married (or female and previous use of family planning were associated with fertility intentions.

**Table 4:** Model 3–Binary logistic regression of migration aspects and social demographic factors with fertility intentions among Street adolescents and youth in Kampala, Uganda, 2019.

**Explanatory factors**	**cOR (95%CI)**	**aOR (95%CI)**
Age in years (12–17 = 0, 18–24 = 1)	2.24 (1.06–4.72)	0.19 (0.01–4.44)
Sex (male = 0, female = 1)	1.04 (0.49–2.21)	0.06 (0.00–2.01)
Education attained (primary = 0, secondary +tertiary = 1)	1.32 (0.54–3.21)	0.08 (0.00–2.0)
Marital status (not married = 0, married = 1)	0.79 (0.32–2.00)	1.50 (0.15–15.10)
Mean household occupants (≤ mean = 0, >mean = 1)	0.44 (0.21–0.97)	0.06 (0.00–1.33)
Religion (other = 0, Christian = 1)	2.55 (1.19–5.48)	0.562 (0.03–12.38)
Residential status (mobile = 0, permanence = 1)	0.83 (0.40–1.76)	2.28 (0.20–24.13)
Sex debut (18 years and above = 1)	1.42 (0.38–5.38)	18.13 (0.59–559.24)
Intra-urban mobility (non-mover = 0, mover = 1)	0.93 (0.41–2.10)	0.34 (0.03–4.38)
Circular migrant (no = 0, yes = 1)	3.06 (0.90–10.45)	32.53 (0.71–1501.21)
Experienced challenges during migration (no = 0, yes = 1)	0.66 (0.29–1.51)	0.20 (0.02–2.05)
Intention to migrate to another town (yes = 1)	1.33 (0.56–3.11)	7.443 (0.52–106.81)
Belongs to a support group (yes = 1)	0.54 (0.23–1.25)	1.736 (0.16–18.80)
Peer/social support (other = 0, friends = 1).	1.13 (0.54–2.38)	1.787 (0.23–13.70)
Migration status (non-migrant = 0, migrant = 1)	1.02 (0.41–2.57)	1.000

## Migration Status as a Predictor of the Three Outcomes

As shown in Tables 2–4, migration status was associated with personal childbearing decision-making, contraceptive, and fertility intentions at the bivariate analysis. After controlling for confounding, the relationship between childbearing decision-making and circular migration and challenges associated with the migration process remained statistically significant. That is, odds of personal childbearing decision making were 77% lower if the street youth had circular migrations compared to their non-circular migrant counterparts. Street adolescents and youth who experience challenges during the migration process were 80% less likely to make personal childbearing decisions (aOR = 0.20, 95%CI 0.05–0.72). Moreover, contraceptive intentions were associated with intra-urban mobility and intentions to migrate. Street adolescents and youth with increased intra-urban mobility were five times more likely to express desires to have children in the future than the less mobile street youth (aOR = 5.33, 95%CI 1.59–17.80). Similarly, street adolescents and youth with intentions to migrate to other urban centers in Uganda were five times more likely to express contraceptive intentions than those without intentions to migrate (aOR = 5.33, 95%CI 1.59–17.80). No significant association was found between fertility intentions and migration status and other social demographic factors in the final multivariate model.

## Discussion

The present study focused on describing, and assessing factors associated with childbearing decision-making, fertility intentions and contraceptive perspectives among urban street adolescents and youth aged 12–24 in Kampala, Uganda. Our study showed that about one in two street adolescents and youth made personal decisions regarding childbearing while 9 in every 10-street youth expressed intentions to have children in the future. Contraceptive intentions were positively associated with intra-urban mobility, intentions to migrate to other towns within the country and self-perceived permanent residence in the capital city. The odds of personal childbearing decision-making were reduced for street youth who were married, lived in a shelter with a population size of more than five people and rural-urban migration and its associated challenges.

The observed fertility desires are higher than what have been reported in most studies in Uganda (16) but almost closer to fertility desires previously reported among HIV clients in, Uganda (34). The higher fertility intentions in this marginalized group of urban youth could be partly explained by the fact that majority of the respondents are very young (72.90% were aged 20 years or younger), majority of whom are not married (83.24%) and therefore still in the prime of their reproductive years. However, given the high youth unemployment rates and the declining trends in fertility rates in the country, positive fertility intentions may tend to be over-optimistic and may or may not necessarily be realized in the future (35). While the fertility intentions among street youth were found to be high, the desire for future contraceptive use was low. This is possibly because most migrant street youth live in urban spaces with their peers who have not attained higher formal education, coupled with low access to family planning services and information in the urban spaces where they live. The high fertility intentions could serve as a major barrier to future contraceptive use among street youth especially the migrants within the urban communities (36).

Sexual and reproductive health decision-making among adolescents and women is influenced by numerous community, relationship and individual level factors (37). Notably, this study provided evidence that social and demographic factors, including migration did not influence fertility intentions of street youth in Kampala. This finding is in contrast with previous studies in which age, educational level, and marital status were associated with fertility intentions (38, 39). Our previous study showed that despite the existence of many health care facilities in Kampala, access to SRH services among street youth were dismal. Within the local study context, many street adolescents and youth in Kampala generally exhibit low literacy levels and high unemployment rates, and hence are not economically empowered (40, 41).

While it is difficult to explain the association between contraceptive intentions and increased intra-urban mobility, it is possible that street youth's mobility within the city spaces could be motivated by the need to access sexual and reproductive services whose access is problematic within the challenging city environment. This finding may suggest that urban residential stability may increase a street youth's desire to access to family planning services over time. It is also possible that those who are mobile are more likely to have unstable relationships and separate with their sexual partners. Separation between migrants and their marital partners has been particularly pronounced in high-fertility and low-contraception settings (36). On the other hand, the observed negative relationship of intention to migrate with the desire to use contraceptives in the future might imply that mobility could potentially inhibit the current and future use of contraceptives. This finding contrasts sharply with findings of a study in Pakistan in which higher mobility outside the household (defined as short-distance movements from the house) was associated with higher intention to use contraceptives (42). This could be explained by differences in socio-cultural contexts and the concept of mobility, which is defined in terms of freedom of movement in some Asian cultures (43). Also, neither of the studies focused on street adolescents or youth within urban environments.

Our study demonstrated that 55% of street youth personal childbearing decision-making which is lower than the figure of 75% reported in a previous study among urban young people in Kampala (41). The observed difference could be attributed the fact that not all urban young people are street adolescents as well as the variation in socio-demographic characteristics especially living arrangements. The odds of personal childbearing decision-making were reduced if the street youth were married, lived in a shelter with a population size of more than five people but significantly increased by four-fold among street youth who belonged to a social support group. Since only a few of the street youth were married, it is possible that they made childbearing decisions as couples as opposed to individual decisions with some degree of peer influence. Previous studies revealed that social networks can also have an influence on women and adolescents' sexual reproductive health behavior and practices of the adolescents (44, 45). These findings provide important insights into need for sexual and reproductive health promotion programmes to be modeled around inter-personal and social interactions and social networks which shaping the childbearing decision-making for the street youth The negative influence of the shelter population size on personal childbearing decision-making may point to the way in which street youth interact with the peers in the new urban environment. It may also suggest that poor housing and living conditions may deprive street youth of confidential sexual activity and personal decision-making due to overcrowding of the shelters which do not permit privacy. Elsewhere, overcrowded family shelters have been found to be as these have been perceived as insecure and stressful for the homeless women to bear children in such an environment (46).

Only half of street youth in our study made personal decisions regarding childbearing and this aspect did not vary with age. This finding contrasts with an earlier study in which women who marry at a young age may have less autonomy to make decisions around modern contraceptive use (47). The relationship between personal childbearing decision-making and migration aspects is interesting although, difficult to explain. However, it could be argued that fact that some street youth who were married could have separated from their sexual partners due to the migration process. It is also possible that street youth are uncertain to make personal decisions to have children due to their increased level of economic vulnerability and the cyclic migrations which sometimes may be challenging. It could be reasoned that the risks associated with migration process such as sexual violence and marital partner separation during movement (31, 48, 49), may negatively impact their wellbeing including their ability to make childbearing choices as having more children may be perceived a burden.

This current study had some strengths and limitations. First, the study used a representative sample of street adolescents and youth who migrated from across all regions of Uganda. Again, the application of a large and representative sample size with adequate power, the use of an electronic questionnaire as well as experienced field enumerators strengthened data quality and the validity of the study findings. Second, the inclusion of known predictors and migration variables with resultant significant associations with outcomes of interest fortified the rigor of the analytical models, and thereby make the findings reliable. In spite of the strengths, caution needs to be applied in interpreting the findings as cross-sectional designs do not permit the establishment of causality of childbearing decision-making, contraceptive and fertility intentions. This study did not assess the relationship of socio-cultural beliefs, perceptions and social norms on street youth's childbearing decision-making, fertility and contractive perspectives, which may warrant further research. In view of the limited literature on street youth's contraceptive and fertility intentions, this study provides the first empirical evidence on the effect of rural-urban migration on personal childbearing decision-making and contraceptive intentions which in turn may predict actual contraceptive adoption by street adolescents and youth. The findings of this study could be generalized to other street youth in high-fertility and low-contraception settings in sub-Saharan Africa.

## Conclusion

This study showed high fertility intentions and low contraceptive desires among street adolescents and youth in Kampala. However, childbearing decision-making, contraceptive and fertility desires are higher among migrant street youth compared to non-migrants. Contraceptive intentions are generally low with slightly more than a half of the street adolescents and youth able to make personal childrearing decision-making. Personal childbearing decision-making was negatively associated with being married, living in overcrowded shelters, rural-urban migration and its associated challenges. Contraceptive intentions were positively associated with intra-urban mobility, intentions to migrate to other towns within the country and self-perceived permanent residence in the city. The factors associated with childbearing decision-making and contraceptive intentions among street adolescents and youth operate mainly at the community, interpersonal and personal levels. The findings have implications in framing future adolescent and SRH policies and strategies in cities while taking into consideration rural-urban migration and intra-urban mobility patterns of street youth. Contraceptive programmes and services to prevent pregnancy and decrease the unmet need for family planning among the street adolescents and youth in Kampala are highly recommended.

## Data Availability Statement

The raw data supporting the conclusions of this article will be made available by the authors, without undue reservation.

## Ethics Statement

The studies involving human participants were reviewed and approved by the Makerere University School of Social Sciences Research and Ethics Committee. Written informed consent to participate in this study was provided by the study participants' guardian/care giver.

## Author Contributions

MB led the conceptualization of the study and its implementation, data analysis, and drafting of the manuscript. CM, PB, and BB contributed to the conceptualization of the study, empirical literature extraction and review, and guided the structuring and technical writing of the manuscript. All authors reviewed and approved the final version of the manuscript.

## Conflict of Interest

The authors declare that the research was conducted in the absence of any commercial or financial relationships that could be construed as a potential conflict of interest.

## Publisher's Note

All claims expressed in this article are solely those of the authors and do not necessarily represent those of their affiliated organizations, or those of the publisher, the editors and the reviewers. Any product that may be evaluated in this article, or claim that may be made by its manufacturer, is not guaranteed or endorsed by the publisher.

## Abbreviations

aOR: adjusted odds ratio
cOR: crude odds ratio
CI: confidence interval
SRH: sexual and reproductive health
HIV: human immunodeficiency virus.

## References

[1] Ekane D,. Fertility trends in Sub Saharan Africa. Stockholm University, Stockholm, Sweden. (2013). Available online at: http://www.diva-portal.org/smash/get/diva2:602495/FULLTEXT01.pdf

[2] World Health Organization. Adolescent Pregnancy WHO. 2020. Available online at: https://www.who.int/news-room/fact-sheets/detail/adolescent-pregnancy (accessed March 6, 2022).

[3] Horschelmann K Van Blerk L. Children, Youth and the City. 1st ed. London, UK: Routledge (2013). p. 1–60.

[4] Lutz W Skirbekk V. How Education Drives Demography and Knowledge Informs Projections. World Popul Hum Cap twenty-first century An Overv. (2013). p. 14–38.

[5] Channon MD Harper S. Educational differentials in the realisation of fertility intentions: is sub-Saharan Africa different? PLoS ONE. (2019) 14:e0219736. 10.1371/journal.pone.0219736 31318943 PMC6638943

[6] Ahinkorah BO. Predictors of unmet need for contraception among adolescent girls and young women in selected high fertility countries in sub-Saharan Africa: a multilevel mixed effects analysis. PLoS ONE. (2020) 15:e0236352. 10.1371/journal.pone.0236352 32760153 PMC7410238

[7] Ahinkorah BO Budu E Aboagye RG Agbaglo E Arthur-Holmes F Adu C. Factors associated with modern contraceptive use among women with no fertility intention in sub-Saharan Africa: evidence from cross-sectional surveys of 29 countries. Contracept Reprod Med. (2021) 6:1–13. 10.1186/s40834-021-00165-6 34332644 PMC8325798

[8] Gelberg L Leake B Lu M Andersen R Wenzel S Morgenstern H. Use of contraceptive methods among homeless women for protection against unwanted pregnancies and sexually transmitted diseases: prior use and willingness to use in the future. Contraception. (2001) 63:277–81. 10.1016/S0010-7824(01)00198-6 11448469

[9] Sserwanja Q Musaba MW Mukunya D. Prevalence and factors associated with modern contraceptives utilization among female adolescents in Uganda. BMC Womens Health. (2021) 21:61. 10.1186/s12905-021-01206-7 33568124 PMC7877106

[10] Uganda Bureau of Statistics (UBOS) ICF. Uganda Demographic and Health Survey 2016. Kampala; Rockville, MD: UBOS; ICF (2016). Available online at: https://dhsprogram.com/pubs/pdf/FR333/FR333.pdf

[11] Litwin LE Makumbi FE Gray R Wawer M Kigozi G Kagaayi J. Impact of availability and use of ART/PMTCT services on fertility desires of previously pregnant women in Rakai, Uganda: a retrospective cohort study. J Acquir Immune Defic Syndr. (2015) 69:377. 10.1097/QAI.0000000000000612 25835605 PMC4506706

[12] Callahan RL Brunie A Mackenzie ACL Wayack-Pambè M Guiella G Kibira SPS. Potential user interest in new long-acting contraceptives: results from a mixed methods study in Burkina Faso and Uganda. PLoS ONE. (2019) 14:e0217333. 10.1371/journal.pone.0217333 31136612 PMC6538161

[13] Ezeh AC Mberu BU Emina JO. Stall in fertility decline in Eastern African countries: regional analysis of patterns, determinants and implications. Philos Trans R Soc B Biol Sci. (2009) 364:2991–3007. 10.1098/rstb.2009.0166 19770151 PMC2781835

[14] Uganda Bureau of Statistics (UBOS). Uganda Bureau of Statistics 2017 Statistical Abstract. Kampala; Uganda Bureau of Statistics (2017).

[15] Population Reference Bureau,. World Population Data Sheet With a Special Focus on Youth. (2017). Available online at: https://www.prb.org/wp-content/uploads/2020/12/World-Population-Data-Sheet-2017-english.pdf

[16] Matovu JKB Makumbi F Wanyenze RK Serwadda D. Determinants of fertility desire among married or cohabiting individuals in Rakai, Uganda: a cross-sectional study. Reprod Health. (2017) 14:2. 10.1186/s12978-016-0272-3 28069056 PMC5223449

[17] ICF UB of S (UBOS) and Uganda Demographic and Health Survey 2016: Key Indicators Report. Kampala; UBOS, and Rockville Maryland (2017).

[18] Blanc AK Tsui AO Croft TN Trevitt JL. Patterns and trends in adolescents'contraceptive use and discontinuation in developing countries and comparisons with adult women. Int Fam Plan Perspect. (2009) 35:63–71. 10.1363/3506309 19620090

[19] Bwambale MF Bukuluki P Moyer CA Van den Borne BHW. Utilisation of sexual and reproductive health services among street children and young adults in Kampala, Uganda: does migration matter? BMC Health Serv Res. (2021) 21:169. 10.1186/s12913-021-06173-1 33622341 PMC7903651

[20] Mirembe S Nzabona A Mushomi JA. Internal youth migration in Uganda: analyzing associates and employment outcomes. Int J Popul Stud. (2019) 5:38–49. 10.18063/ijps.v5i1.969

[21] Brockerhoff M Yang X. Impact of migration on fertility in sub-Saharan Africa. Soc Biol. (1994) 41:19–43. 10.1080/19485565.1994.9988857 7973839

[22] Uganda Bureau of Statistics (UBOS) ICF. Uganda Demographic and Health Survey 2016. Kampala; Rockville, MD: UBOS; ICF (2016). Available online at: https://dhsprogram.com/pubs/pdf/FR333/FR333.pdf

[23] Sarnak DO Wood SN Zimmerman LA Karp C Makumbi F Kibira SPS. The role of partner influence in contraceptive adoption, discontinuation, and switching in a nationally representative cohort of Ugandan women. PLoS ONE. (2021) 16:e0238662. 10.1371/journal.pone.0238662 33434205 PMC7802956

[24] Tusubira AK Kibira SPS Makumbi FE. Modern contraceptive use among postpartum women living with HIV attending mother baby care points in Kabarole District, Uganda. BMC Womens Health. (2020) 20:78. 10.1186/s12905-020-00944-4 32321480 PMC7178756

[25] Song X Grilo SA Mathur S Lutalo T Ssekubugu R Nalugoda F. Differential Impacts of HIV status on short-term fertility desires among couples in Rakai, Uganda. PLoS ONE. (2019) 14:e0210935. 10.1371/journal.pone.0210935 30677068 PMC6345474

[26] Fikree FF Lane C Simon C Hainsworth G MacDonald P. Making good on a call to expand method choice for young people - turning rhetoric into reality for addressing sustainable development goal three. Reprod Health. (2017) 14:53. 10.1186/s12978-017-0313-6 28399923 PMC5387301

[27] Aufseeser D. Street children and everyday violence. In: Skelton T, Harker C, Horschelmann K, editors. Conflict, Violence and Peace. Geographies of Children and Young People, Vol. 11. Singapore: Springer (2017). p. 109–27. 10.1007/978-981-287-038-4_31

[28] Kampala Capital City Authority,. Kampala Capital City Authority (KCCA) Strategic Plan 2014/15-2018/19: Laying the Foundation for Kampala City Transformation (2014). Available online at: https://www.kcca.go.ug/uploads/KCCA_STRATEGI_PLAN_2015-2016.pdf

[29] Bwambale MF Bukuluki P Moyer CA Borne BHWVD. Demographic and behavioural drivers of intra-urban mobility of migrant street children and youth in Kampala, Uganda. PLoS ONE. (2021) 16:e0247156. 10.1371/journal.pone.0247156 33600461 PMC7891785

[30] Embleton L Nyandat J Ayuku D Sang E Kamanda A Ayaya S. Sexual behavior among orphaned adolescents in western Kenya: a comparison of institutional-and family-based care settings. J Adolesc Heal. (2017) 60:417–24. 10.1016/j.jadohealth.2016.11.015 28110864 PMC5389113

[31] Sznajder KK Winchester MS Biney AAE Dodoo ND Letsa D Dodoo FN-A. The migration experience and differential risks to sexual and reproductive health in Ghana. Heal Educ Behav. (2020) 47:718–27. 10.1177/1090198120939492 32639174

[32] Sanga SA. Intra-urban residential mobility and tenants' workplace choices in Kinondoni municipality. Habitat Int. (2015) 49:45–55. 10.1016/j.habitatint.2015.05.006

[33] Andreasen MH Agergaard J. Residential mobility and homeownership in Dar es Salaam. Popul Dev Rev. (2016) 42:95–110. 10.1111/j.1728-4457.2016.00104.x

[34] Wagner GJ Mindry D Hurley EA Beyeza-Kashesya J Gwokyalya V Finocchario-Kessler S. Reproductive intentions and corresponding use of safer conception methods and contraception among Ugandan HIV clients in serodiscordant relationships. BMC Public Health. (2021) 21:1–14. 10.1186/s12889-021-10163-7 33468072 PMC7814634

[35] Vidal S Huinink J Feldhaus M. Fertility intentions and residential relocations. Demography. (2017) 54:1305–30. 10.1007/s13524-017-0592-0 28699099

[36] Cau BM. Female migration, local context and contraception use in urban Mozambique. Afr J Reprod Health. (2016) 20:52–61. 10.29063/ajrh2016/v20i1.5 29553177

[37] Svanemyr J Amin A Robles OJ Greene ME. Creating an enabling environment for adolescent sexual and reproductive health: a framework and promising approaches. J Adolesc Heal. (2015) 56(1 Suppl):S7–14. 10.1016/j.jadohealth.2014.09.011 25528980

[38] Testa MR. On the positive correlation between education and fertility intentions in Europe: individual-and country-level evidence. Adv Life Course Res. (2014) 21:28–42. 10.1016/j.alcr.2014.01.005 26047540 PMC4477715

[39] Mussino E Gabrielli G Ortensi LE Strozza S. Fertility intentions within a 3-Year time frame: A comparison between migrant and native Italian women. J Int Migrat Integrat. (2021) 1–28. 10.1007/s12134-020-00800-2

[40] Kamara JK Namugambe BM Egessa R Kamanga G Renzaho AMN. The socioeconomic and sexual health status of young people living in urban slum areas of Kampala, Uganda. J Urban Health. (2019) 96:616–31. 10.1007/s11524-019-00347-3 30790124 PMC6890897

[41] Renzaho AMN Kamara JK Georgeou N Kamanga G. Sexual, reproductive health needs, and rights of young people in Slum Areas of Kampala, Uganda: a cross sectional study. PLoS ONE. (2017) 12:e0169721. 10.1371/journal.pone.0169721 28107371 PMC5249247

[42] Hamid S Stephenson R Rubenson B. Marriage decision making, spousal communication, and reproductive health among married youth in Pakistan. Glob Health Action. (2011) 4:5079. 10.3402/gha.v4i0.5079 21253456 PMC3023879

[43] Riyami A Afifi M. Women empowerment and marital fertility in Oman. J Egypt Public Health Assoc. (2003) 78:55–72. Available online at: http://europepmc.org/abstract/MED/17219911 17219911

[44] Asrese K Mekonnen A. Social network correlates of risky sexual behavior among adolescents in Bahir Dar and Mecha Districts, North West Ethiopia: an institution-based study. Reprod Health. (2018) 15:1–8. 10.1186/s12978-018-0505-8 29642938 PMC5896065

[45] Osur J Orago A Mwanzo I Bukusi E. Social networks and decision making for clandestine unsafe abortions: evidence from Kenya. Afr J Reprod Health. (2015) 9:34–43. 26103693

[46] Kennedy S Grewal MPHM Roberts EM Steinauer J Dehlendorf C. A qualitative study of pregnancy intention and the use of contraception among homeless women with children. J Health Care Poor Underserved. (2014) 25:757. 10.1353/hpu.2014.0079 24858884 PMC4232303

[47] McClendon KA McDougal L Ayyaluru S Belayneh Y Sinha A Silverman JG. Intersections of girl child marriage and family planning beliefs and use: qualitative findings from Ethiopia and India. Cult Health Sex. (2018) 20:799–814. 10.1080/13691058.2017.1383513 29043910

[48] Keygnaert I Dialmy A Manço A Keygnaert J Vettenburg N Roelens K. Sexual violence and sub-Saharan migrants in Morocco: a community-based participatory assessment using respondent driven sampling. Global Health. (2014) 10:1–16. 10.1186/1744-8603-10-32 24885537 PMC4122073

[49] Marrujo OTR. Women, migration, and sexual violence: Lessons from Mexico's borders. In: Human Rights Along the US-Mexico Border: Gendered Violence and Insecurity. (2009). p. 31–47.

